# Lipoprotein(a) and Risk of Incident Atherosclerotic Cardiovascular Disease: Impact of High-Sensitivity C-Reactive Protein and Risk Variability Among Human Clinical Subgroups

**DOI:** 10.3390/nu17081324

**Published:** 2025-04-11

**Authors:** Ron C. Hoogeveen, Margaret R. Diffenderfer, Elise Lim, Ching-Ti Liu, Hiroaki Ikezaki, Weihua Guan, Michael Y. Tsai, Christie M. Ballantyne

**Affiliations:** 1 Section of Cardiovascular Research, Department of Medicine, Baylor College of Medicine, Houston, TX 77030, USA; cmb@bcm.edu; 2 Cardiovascular Nutrition Laboratory, Jean Mayer USDA Human Nutrition Research Center on Aging at Tufts University, Boston, MA 02111, USA; ikezaki.hiroaki.472@m.kyushu-u.ac.jp; 3 Boston Heart Diagnostics, Framingham, MA 01702, USA; 4 Department of Biostatistics, Boston University School of Public Health, Boston, MA 02118, USA; elise625@gmail.com (E.L.); ctliu@bu.edu (C.-T.L.); 5 The Framingham Heart Study, National Heart, Lung, and Blood Institute, Framingham, MA 01702, USA; 6 Department of General Internal Medicine, Kyushu University Hospital, Fukuoka 812-8582, Japan; 7 Division of Biostatistics and Health Data Science, School of Public Health, University of Minnesota, Minneapolis, MN 55414, USA; wguan@umn.edu; 8 Department of Laboratory Medicine and Pathology, University of Minnesota Medical School, Minneapolis, MN 55455, USA; tsaix001@umn.edu; 9 Section of Cardiology, Department of Medicine, Baylor College of Medicine, Houston, TX 77030, USA; 10 Center for Cardiovascular Disease Prevention, Debakey Heart and Vascular Disease Center, Houston, TX 77030, USA

**Keywords:** Lp(a), cardiovascular disease, CRP, diabetes

## Abstract

**Background/Objectives:** Elevated lipoprotein(a) [Lp(a)] is associated with increased incidence of atherosclerotic cardiovascular disease (ASCVD). We aimed to assess the utility of Lp(a) as an ASCVD risk-enhancing factor, as recommended by the 2019 ACC/AHA guidelines on ASCVD primary prevention, and to determine whether C-reactive protein (CRP) modifies the association of elevated Lp(a) with ASCVD risk. **Methods:** Lp(a), high sensitivity CRP (hs-CRP), and other ASCVD risk factors, including blood lipids, blood pressure, diabetes status, body weight and height, and smoking, were measured in 15,933 participants (median age 61.7 years with 25th–75th percentiles 57–68 years, 56.7% female, 19.7% Black, free of ASCVD at baseline) in the Atherosclerosis Risk in Communities Study, Framingham Offspring Study, and Multi-Ethnic Study of Atherosclerosis. Participants were followed for 10 years for incident ASCVD (coronary heart disease (CHD) or stroke) and CHD (including angioplasty and/or coronary artery bypass but minus stroke). These endpoints occurred in 9.7% and 7.4% of subjects, respectively. **Results:** Compared with the lowest Lp(a) category (<10 mg/dL), the highest Lp(a) category (≥50 mg/dL) carried a significantly increased incidence of ASCVD (hazard ratio [HR] = 1.31; 95% confidence interval [CI] 1.15–1.50; *p* < 0.001) and CHD (HR = 1.49; 95%CI 1.27–1.75; *p* < 0.001). The association of elevated Lp(a) with incident ASCVD was stronger in males and non-Black individuals and was independent of diabetes status. Lp(a) levels ≥ 50 mg/dL predicted the 10-year ASCVD risk for those at intermediate risk (≥7.5%, HR = 1.32; 95%CI 1.15–1.52; *p* < 0.001). There was a significant interaction between Lp(a) and hs-CRP; individuals with concomitant elevated levels of Lp(a) and hs-CRP had the highest ASCVD risk. **Conclusions:** Elevated Lp(a) levels were associated with increased ASCVD risk, particularly in individuals with concomitantly elevated hs-CRP levels and those at intermediate 10-year ASCVD risk.

## 1. Introduction

Lipoprotein(a) [Lp(a)] is an atherogenic lipoprotein composed of a low-density lipoprotein (LDL)-like moiety with a unique glycoprotein, apolipoprotein (a) [apo(a)], that is covalently bound to its apoB-100 moiety [1]. It is generally believed that in addition to its atherogenic properties, Lp(a) has prothrombotic and proinflammatory properties.

Over the past two decades, prospective studies, Mendelian randomization, and genome-wide association studies have shown that elevated Lp(a) is likely to be a causal risk factor for atherosclerotic cardiovascular disease (ASCVD) [2,3,4,5]. Circulating levels of Lp(a) are primarily determined by heredity, particularly genetic variability at the *LPA* gene locus [6]. Novel antisense oligonucleotides and small interfering RNA therapies have shown potent Lp(a)-lowering efficacy [7,8]. The recent American Heart Association/American College of Cardiology guidelines on the primary prevention of ASCVD recommend the use of Lp(a) as a risk-enhancing factor that can help to refine risk estimates in individuals aged 40–75 years at borderline or intermediate risk of ASCVD [9].

Low-grade chronic inflammation plays a key role in the development of atherosclerosis, and recent randomized controlled trials have demonstrated that specific anti-inflammatory therapies improve cardiovascular outcomes [10,11]. As an inflammatory biomarker, high-sensitivity C-reactive protein (hs-CRP) has been the most validated ASCVD risk predictor related to inflammation; the 2019 American Heart Association/American College of Cardiology guidelines on the primary prevention of ASCVD recommend the measurement of hs-CRP as a risk-enhancing factor [9]. Recently, an analysis from the Multi-Ethnic Study of Atherosclerosis (MESA) showed that elevated Lp(a) levels were associated with increased ASCVD risk only in those individuals with concomitant elevated hs-CRP levels (≥2 mg/L), suggesting that Lp(a)-associated ASCVD risk is exacerbated in a proinflammatory milieu [12].

In the current study, we examined the association between Lp(a) and ASCVD events in non-Black and Black adults in the combined cohorts of the Atherosclerosis Risk in Communities Study (ARIC), the Framingham Offspring Study (FOS), and MESA. Furthermore, we evaluated the utility of Lp(a) as an ASCVD risk-enhancing factor and whether hs-CRP modified the association of elevated Lp(a) with ASCVD risk. Our overall hypothesis was that Lp(a) can be used as an ASCVD risk-enhancing factor and that elevated Lp(a) levels predict incident ASCVD risk even in individuals that do not have elevated hs-CRP levels.

## 2. Materials and Methods

### 2.1. Study Population

Study subjects were participants in ARIC visit 4 (1996–1998), FOS cycle 6 (1995–1998), or the MESA baseline exam (2000–2002). The current analyses include 15,933 subjects (median age 61.7 [57–68] years); of these, 9038 (56.7%) were female, and 3141 (19.7%) were Black people. Non-Black people (12,792, 80.3%) included study participants of European (11,070, 69.5%), Hispanic/Latino (973, 6.1%), or Asian (528, 3.3%) ancestry (Supplemental Table S1). All subjects were followed for 10 years for the development of ASCVD. All subjects were required to meet the following criteria: (1) be free of inclusive ASCVD (coronary heart disease [CHD], including angioplasty and/or coronary artery bypass, and stroke), (2) have frozen plasma available from blood sampled after an overnight fast, (3) have had a baseline history and physical examination (including measurement of blood pressure, height, and weight) as part of their participation in the study, and (4) have follow-up data available.

All subjects provided information about their past medical history and use of medications and supplements. Hypertension was defined as a blood pressure measurement >140 mnHg systolic or 90 mmHg diastolic, or being on medications for hypertension. Diabetes was defined as a fasting glucose level >125 mg/dL or being on medications for diabetes. Smoking was defined as cigarette smoking within the past year. At baseline, 6.7% of the subjects were taking lipid-lowering medications. All studies were conducted in accordance with the Declaration of Helsinki, and all participating study centers approved (including field centers). Written informed consent was obtained from all subjects involved in the three studies.

### 2.2. Laboratory Measurements

Fasting plasma samples that were stored at −80 °C and had never thawed were used for the analysis. Plasma levels of total cholesterol, triglycerides (TG), and high-density lipoprotein cholesterol (HDL-C) were determined by standard enzymatic methods, as previously described [13]. Lp(a) was measured using a commercially available automated immunoturbidimetric assay (Denka Seiken Co., Ltd., Tokyo, Japan) insensitive to apo(a) isoform variations [14]. Non-HDL-C was calculated as the total cholesterol–HDL-C. LDL-cholesterol (LDL-C) was calculated using the Friedewald formula as total cholesterol–HDL-C–TG/5 [15]. All laboratory data were generated at the central laboratories of each study (for ARIC, at Baylor College of Medicine; for FOS at Tufts University; and for MESA at the University of Minnesota) using numbered samples in a blinded fashion.

### 2.3. Outcomes

For prospective inclusive ASCVD endpoints in this analysis, we used the following criteria: the development of myocardial infarction (recognized with or without diagnostic electrocardiogram, but including cardiac biomarkers and history or recognized at the time of autopsy), coronary revascularization (angioplasty or coronary artery bypass grafting), stroke (atherothrombotic brain infarction, cerebral embolism, definite or other cardiovascular accident, intracerebral hemorrhage, subarachnoid hemorrhage), and death from either myocardial infarction or stroke (sudden death from CHD, death from cerebrovascular accident, death from other cardiovascular disease). For CHD criteria, we used ASCVD criteria and excluded all subjects who had experienced a stroke. For ischemic stroke criteria, we excluded all subjects who developed CHD and included only those who had an ischemic stroke, excluding subjects that had an intracerebral hemorrhage and/or a subarachnoid hemorrhage. Only the first event over a follow-up time of up to 10 years was used in the analysis.

### 2.4. Statistical Analysis

Statistical analyses were performed using R software, version 3.6.0 (R Foundation, Vienna, Austria). *p* < 0.05 was considered statistically significant.

Data from all three studies were pooled and analyzed in a blinded fashion. Continuous variables were expressed as median values with 25th–75th percentiles, and categorical variables were expressed as frequencies and percentages. TG, Lp(a), and hs-CRP levels were not normally distributed and were log-transformed prior to all statistical analyses. Lp(a) concentrations were assessed as continuous (per log unit increase) and as categorical (<10, 10–<30, 30–<50, ≥50 mg/dL) variables for the association with the risk of incident ASCVD, CHD, and ischemic stroke events. Incidence rates (per 1000 person-years) and 10-year absolute risk of ASCVD events were also calculated. *p*-value was calculated for the linear trend of risk across the Lp(a) categories.

Using Cox proportional-hazards regression, the hazard ratios (HRs) for incident ASCVD events were calculated for Lp(a) categories, with the lowest category as reference. Data were adjusted for age, sex, and race (model 1), and additionally for smoking status, blood pressure, blood pressure medications, total cholesterol, and HDL-C (pooled cohort equation [PCE] variables) and cholesterol-lowering medications (model 2). The risk of incident ASCVD was also compared among 4 risk groups classified by normal hs-CRP (<2.0 mg/L) or elevated hs-CRP (≥2.0 mg/L) and normal Lp(a) (<50 mg/dL) or elevated Lp(a) (≥50 mg/dL). A test of multiplicative interaction between Lp(a) and hs-CRP was performed.

## 3. Results

### 3.1. Baseline Characteristics

The median age of the study population was 61.7 years (±11 years). Table 1 shows the baseline characteristics of the study participants stratified by Lp(a) quintiles. Elevated Lp(a) levels were more frequent among females than males and among Black people compared with non-Black people. Those individuals with Lp(a) levels in the highest quintile had greater median plasma levels of total cholesterol, LDL-C, and HDL-C, but lower TG levels. The percentage of participants taking anti-diabetes and cholesterol-lowering drugs increased with increasing Lp(a) levels.

### 3.2. Demographics and ASCVD Outcomes

Supplemental Table S1 shows the event outcomes by sex and ethnic demographics. Of the 15,933 subjects studied, 1548 (9.72%) had an ASCVD event, 1168 (7.33%) had a CHD event, and 335 (2.09%) had an ischemic stroke over the 10-year follow-up period. As shown in Table 2, the hazards for ASCVD, CHD, or ischemic stroke in females were 59%, 66%, and 43%, respectively, lower than those observed for males (all *p* < 0.0001). Black people had a similar hazard for ASCVD, 17% lower risk of CHD (*p* < 0.05), and 72% higher risk of ischemic stroke (*p* < 0.0001), as compared to non-Black people.

Table 2 shows that all standard risk factors and other parameters were significantly different at baseline between subjects who developed ASCVD and CHD versus those who did not develop these endpoints over the follow-up period. Both Lp(a) levels and hs-CRP levels were significantly higher in subjects with incident ASCVD and CHD versus those who did not have cardiovascular events.

### 3.3. Lp(a) and ASCVD Risk in Multivariate Analysis

**Figure 1:**
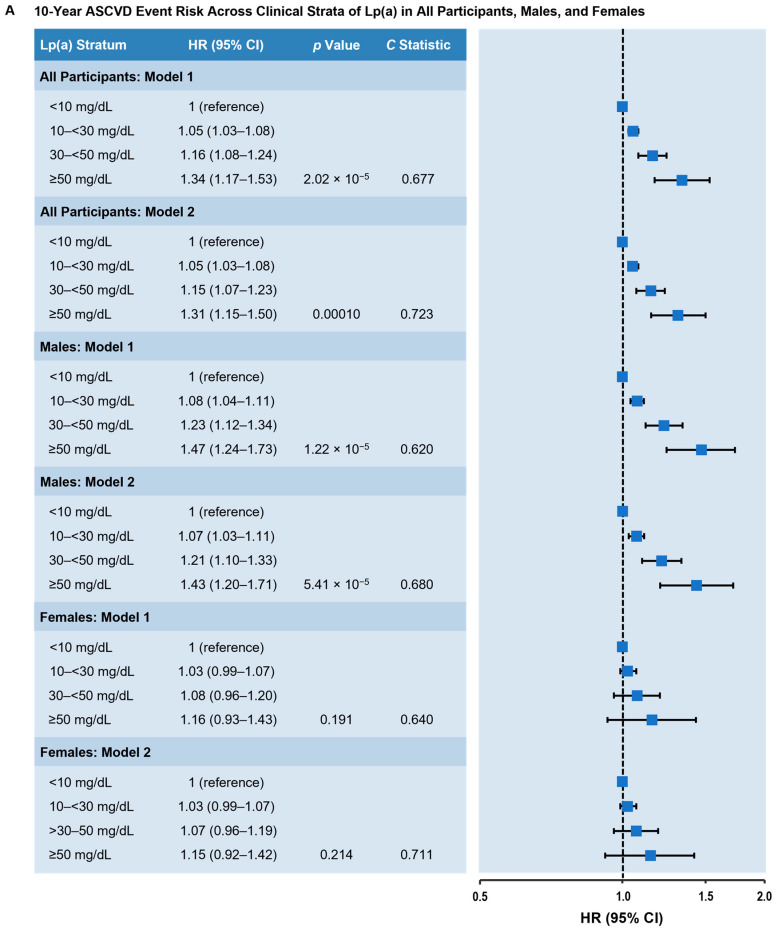

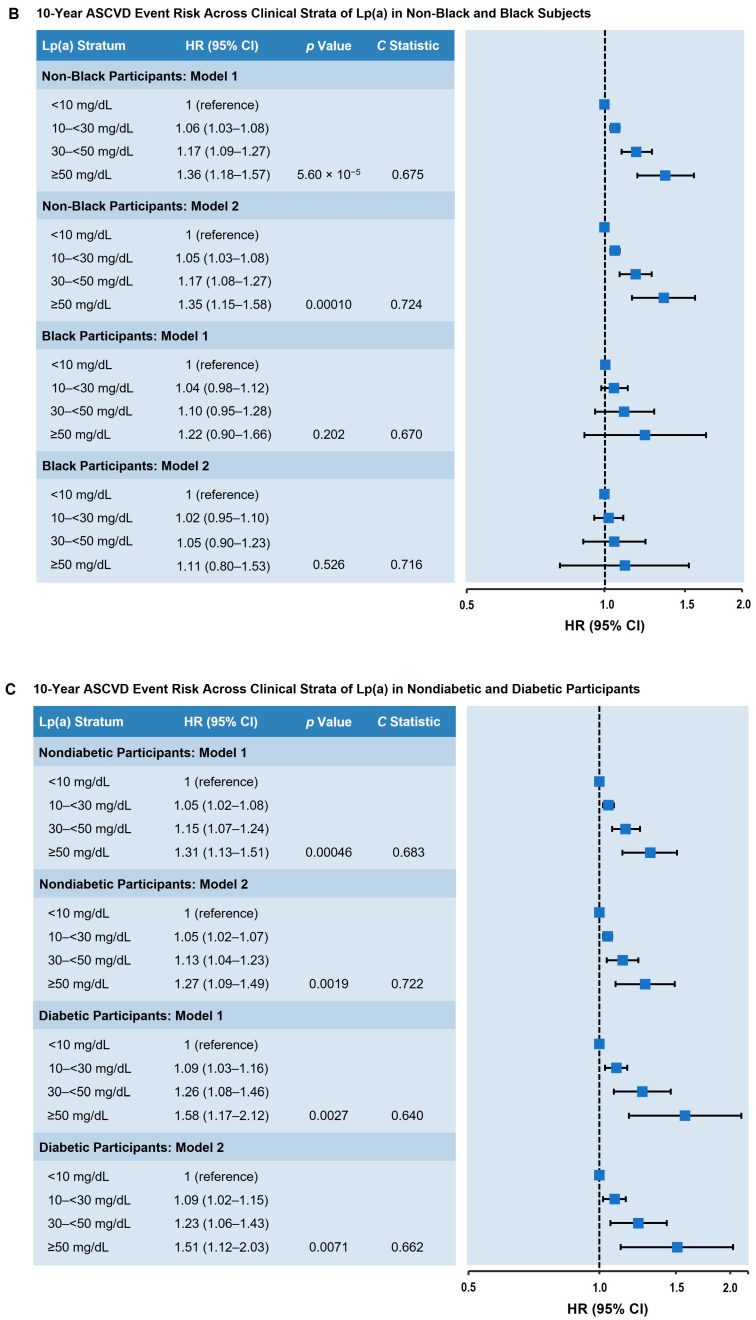
Adjusted 10-year inclusive ASCVD event risk across clinical strata of Lp(a). (**A**) All participants, males, and females. (**B**) Non-Black and Black participants. (**C**) Non-diabetic and diabetic participants. Model 1: Adjusted for age, sex, and race. Model 2: Adjusted for age, sex, race, diabetes status, hypertension, hypertension treatment, smoking status, total cholesterol, high-density lipoprotein cholesterol, and cholesterol-lowering medication use. Lp(a) values were classified according to prespecified clinical strata. The HR (95% CI) is shown in comparison to the Lp(a) < 10 mg/dL stratum (reference). *p* value represents trend across the categories; the *C* statistic represents the probably of an event across the categories. Abbreviations: ASCVD, atherosclerotic cardiovascular disease; CI, confidence interval; HR, hazard ratio; Lp(a), lipoprotein(a).

**Figure 2:**
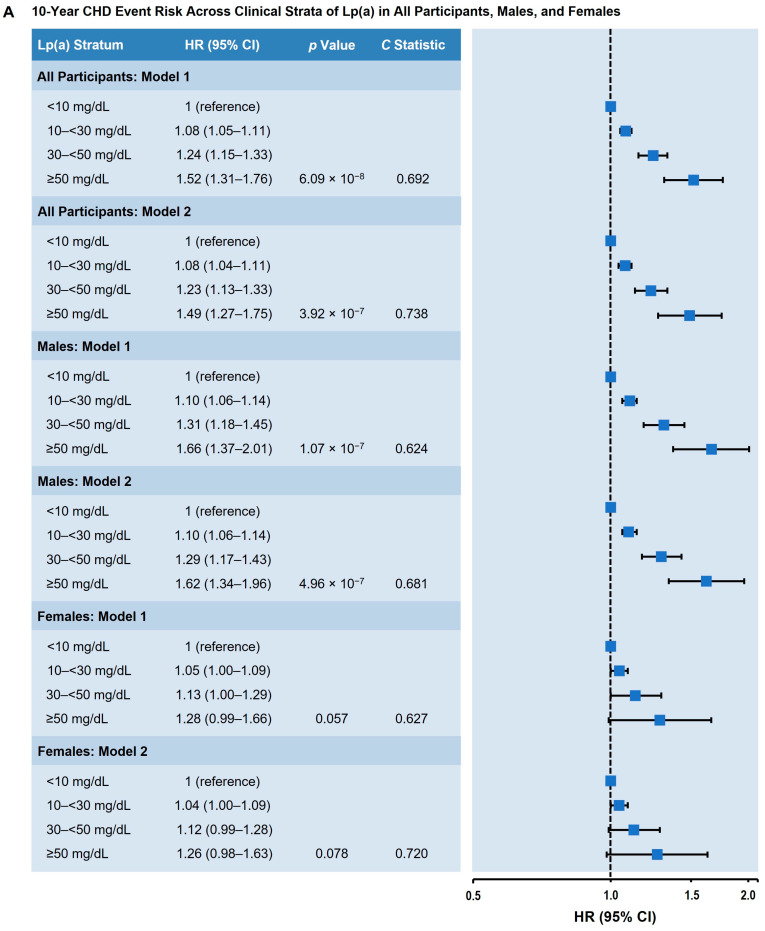

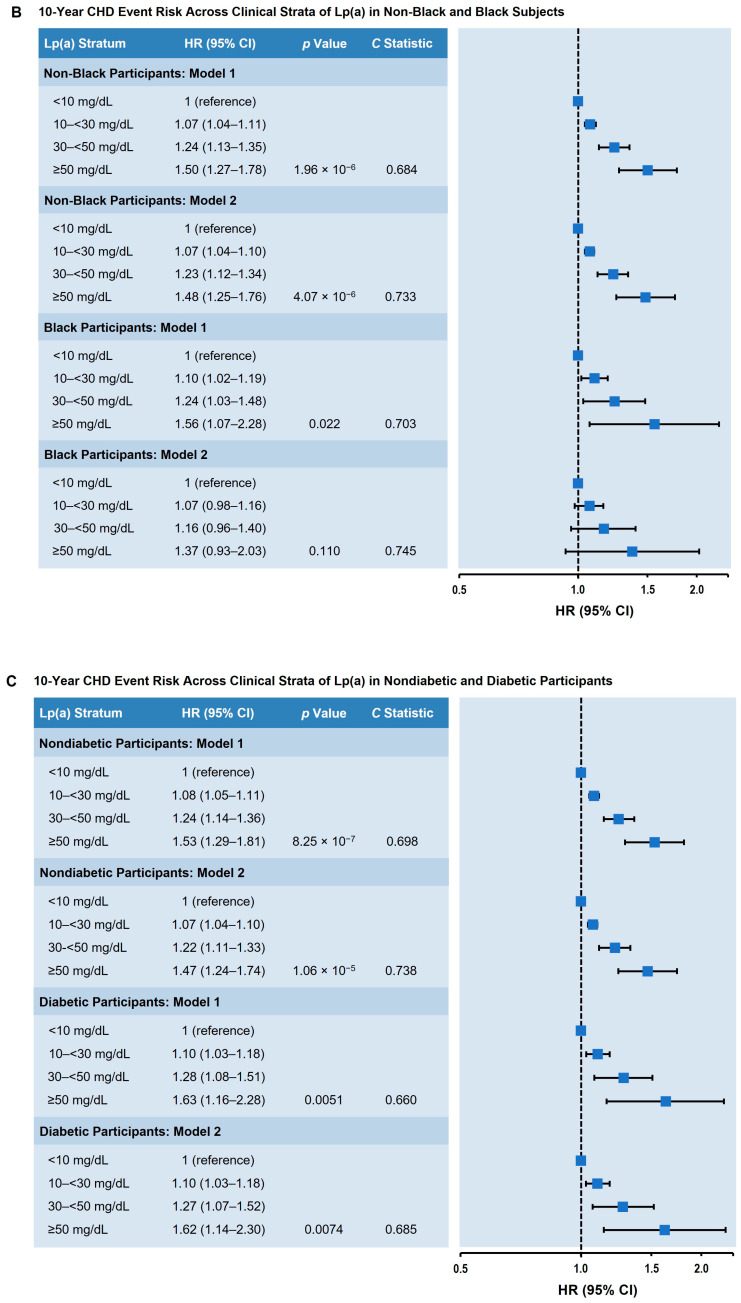
Adjusted 10-year CHD risk across clinical strata of Lp(a). (**A**) All participants, males, and females. (**B**) Non-Black and Black participants. (**C**) Non-diabetic and diabetic participants. Model 1: Adjusted for age, sex, and race. Model 2: Adjusted for age, sex, race, diabetes status, hypertension, hypertension treatment, smoking status, total cholesterol, high-density lipoprotein cholesterol, and cholesterol-lowering medication use. Lp(a) values were classified according to prespecified clinical strata. The HR (95% CI) is shown in comparison to the Lp(a) < 10 mg/dL stratum (reference). *p* value represents trend across the categories; the *C* statistic represents the probably of an event across the categories. Abbreviations: CHD, coronary heart disease; CI, confidence interval; HR, hazard ratio; Lp(a), lipoprotein(a).

We used Lp(a) < 10 mg/dL as the lowest Lp(a) risk category because 10 mg/dL approximately resembled the median Lp(a) concentration in individuals who did not experience ASCVD events. When modeled as a categorical variable and comparing the highest to lowest Lp(a) categories (≥50 mg/dL vs. <10 mg/dL), the highest Lp(a) level was significantly associated with greater incident ASCVD in fully adjusted models (HR = 1.31; 95% confidence interval [CI] 1.15–1.50; *p* < 0.001; Figure 1A). The association of elevated Lp(a) levels with incident ASCVD was stronger in males (HR = 1.43; 95% CI 1.20–1.71; *p* < 0.001; Figure 1A) and non-Black subjects (HR = 1.35; 95% CI 1.15–1.58; *p* < 0.001; Figure 1B) and not statistically significant in females (HR = 1.15; 95% CI 0.92–1.42; *p* = 0.711; Figure 1A) and Black subjects (HR = 1.11; 95% CI 0.80–1.53; *p* = 0.526; Figure 1B). Elevated Lp(a) levels were significantly associated with 10-year inclusive ASCVD risk in non-diabetic (HR = 1.27; 95% CI 1.09–1.49; *p* = 0.002) and diabetic subjects (HR = 1.51; 95% CI 1.12–2.03; *p* = 0.007) (Figure 1C). Similarly, elevated Lp(a) was associated with 10-year incident CHD risk (HR = 1.49; 95% CI 1.27–1.75; *p* < 0.001; Figure 2A), with stronger associations in males and non-Black subjects (Figure 2A,B) and a greater risk of incident CHD in diabetics compared with non-diabetic participants (Figure 2C).

We did not find any significant associations of Lp(a) with incident ischemic stroke events (HR_unadjusted_ = 1.02; 95% CI 0.91–1.14; *p* = 0.76), and Lp(a) levels were not different in subjects with ischemic stroke vs. non-cases (14.2 mg/dL [5.8–38.2] vs. 14.5 mg/dL [6.0–38.3], respectively; *p* > 0.05). These data indicate that the association of elevated Lp(a) levels with incident ASCVD events was mostly driven by CHD events rather than by ischemic stroke events.

### 3.4. Utility of Lp(a) as a Risk-Enhancing Factor by 10-Year ASCVD Risk Categories

**Figure 3:**
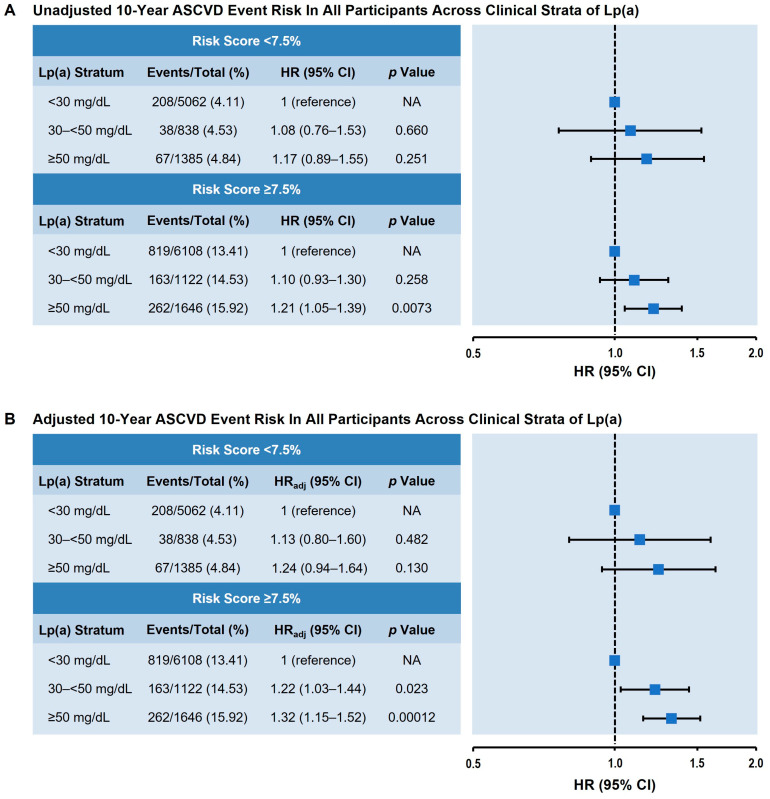
The 10-year ASCVD event risk of all participants across clinical strata of Lp(a) dichotomized by pooled cohort equation risk score. (**A**) Unadjusted event risk. (**B**) Adjusted event risk, adjusted for age, sex, race, systolic blood pressure, hypertension treatment, total cholesterol, smoking status, and high-density lipoprotein cholesterol. Lp(a) values were classified according to prespecified clinical strata. HR (95% CI), HR_adj_, and *p* value are shown in comparison to the Lp(a) < 30 mg/dL stratum (reference). Abbreviations: ASCVD, atherosclerotic cardiovascular disease; CI, confidence interval; HR, hazard ratio; HR_adj_, adjusted hazard ratio; Lp(a), lipoprotein(a).

As shown in Figure 3, we investigated Lp(a)-associated ASCVD risk according to pooled cohort equation-calculated risk score categories (i.e., “borderline risk” < 7.5% 10-year ASCVD risk and “intermediate risk” ≥ 7.5% 10-year ASCVD risk). Lp(a) levels ≥ 50 mg/dL were associated with increased 10-year ASCVD risk for those at intermediate risk (≥7.5%; HR = 1.32; 95% CI 1.15–1.52; *p* < 0.001). Although Lp(a) levels ≥ 50 mg/dL were associated with increased 10-year ASCVD risk in those at borderline risk (<7.5%), the association was not statistically significant (HR = 1.24; 95% CI 0.94–1.64; *p* = 0.13) (Figure 3B).

### 3.5. Impact of hs-CRP on Lp(a) Associated ASCVD Risk

**Figure 4:**
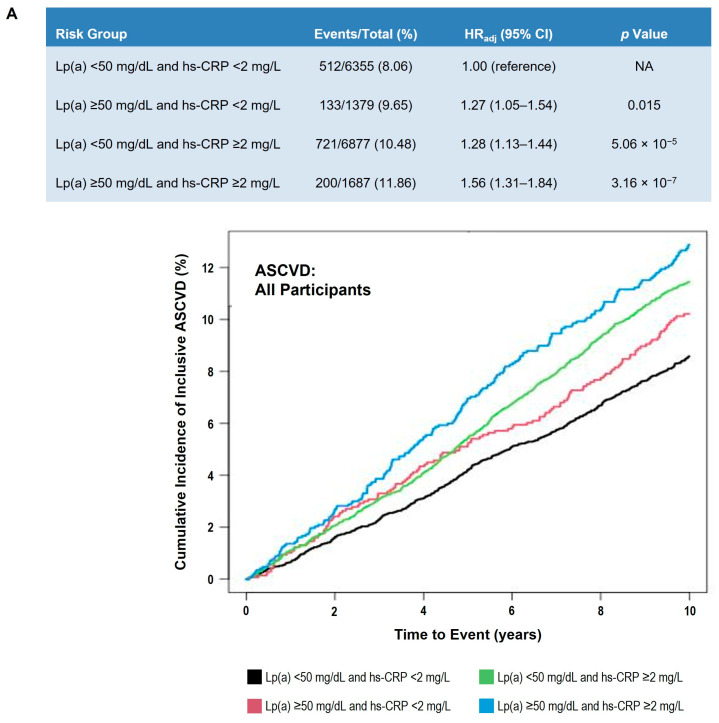

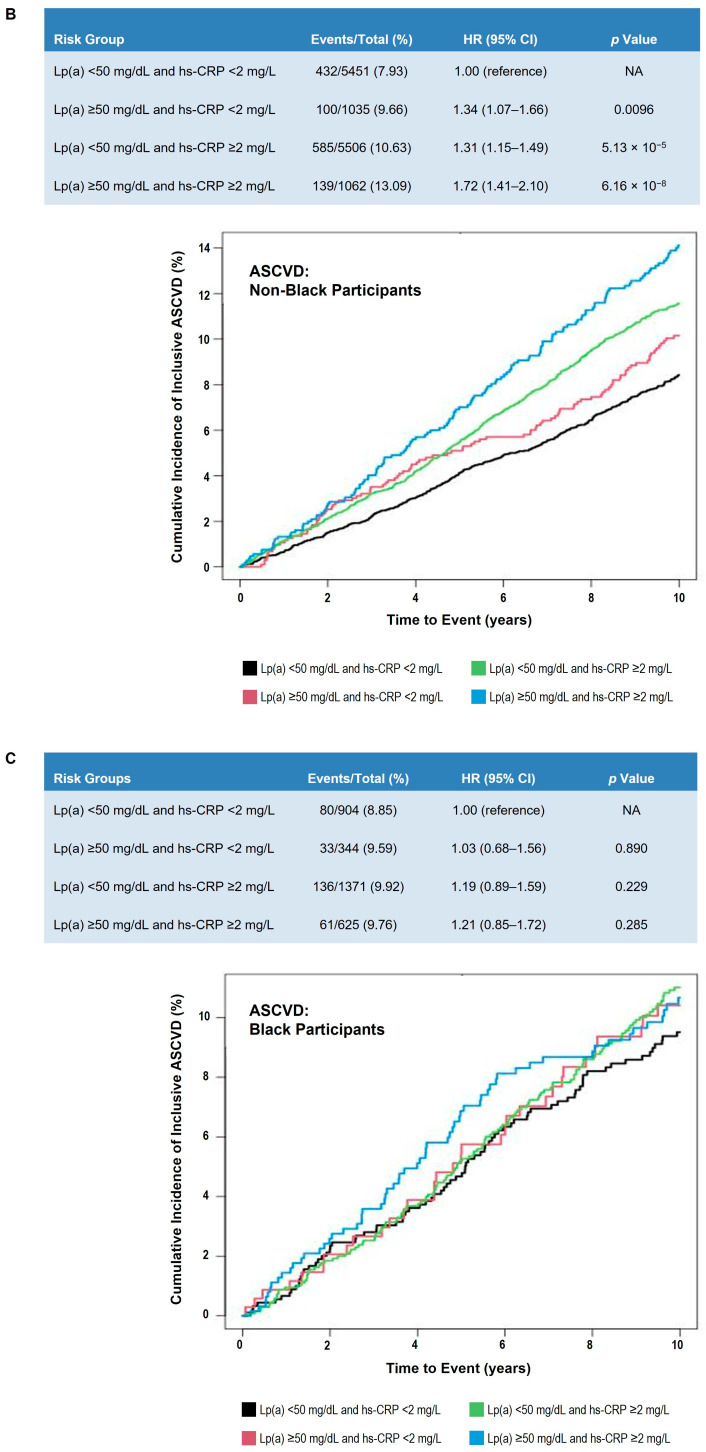
Adjusted 10-year ASCVD event risk in participants classified by Lp(a) and hs-CRP levels. (**A**) All participants. (**B**) Non-Black participants. (**C**) Black participants. Table shows number of events, HR_adj_, and *p* value by Lp(a) and hs-CRP clinical strata. Cox proportional hazards model was adjusted for age, sex, race (Panel A only), systolic blood pressure, hypertension treatment, smoking status, diabetes status, total cholesterol, high-density lipoprotein cholesterol, and cholesterol-lowering medication use. Lp(a) < 50 mg/dL and hs-CRP < 2 mg/L was used as the reference group. Graph presents Kaplan–Meier curves of cumulative incidence of events by prespecified Lp(a) and hs-CRP clinical strata. Black line indicates Lp(a) < 50 mg/dL and hs-CRP < 2 mg/L; red line shows Lp(a) ≥ 50 mg/dL and hs-CRP < 2 mg/L; green line shows Lp(a) < 50 mg/dL and hs-CRP ≥ 2 mg/L; blue line shows ≥50 mg/dL and hs-CRP ≥ 2 mg/dL. Abbreviations: ASCVD, atherosclerotic cardiovascular disease; CHD, coronary heart disease; CI, confidence interval; HR, hazard ratio; HR_adj_, adjusted hazard ratio; hs-CRP, high sensitivity C-reactive protein; Lp(a), lipoprotein(a).

We found a significant interaction between Lp(a) and hs-CRP (P_interaction_ log Lp(a) × log hs-CRP < 0.001 and P_interaction_ Lp(a) (<50 or ≥50 mg/dL) × hs-CRP (<2 or ≥2 mg/L) < 0.001) and investigated the impact of hs-CRP levels on Lp(a)-associated ASCVD risk by stratifying subjects according to hs-CRP categories (“normal hs-CRP” < 2 mg/L vs. “high hs-CRP” ≥ 2 mg/L) and Lp(a) categories (Lp(a) < 50 mg/dL vs. Lp(a) ≥ 50 mg/dL) (Figure 4). Those individuals with concomitant elevated levels of Lp(a) and hs-CRP were at the highest ASCVD risk (HR = 1.56; 95% CI 1.31–1.84; *p* < 0.0001). However, elevated Lp(a) levels were also associated with increased ASCVD risk in individuals with normal (<2 mg/L) hs-CRP levels (HR = 1.27; 95% CI 1.05–1.54; *p* < 0.015) (Figure 4A). We found similar results in non-Black subjects (Figure 4B), but no significant effect of hs-CRP on the association of Lp(a) with incident ASCVD in Black subjects (Figure 4C).

**Figure 5:**
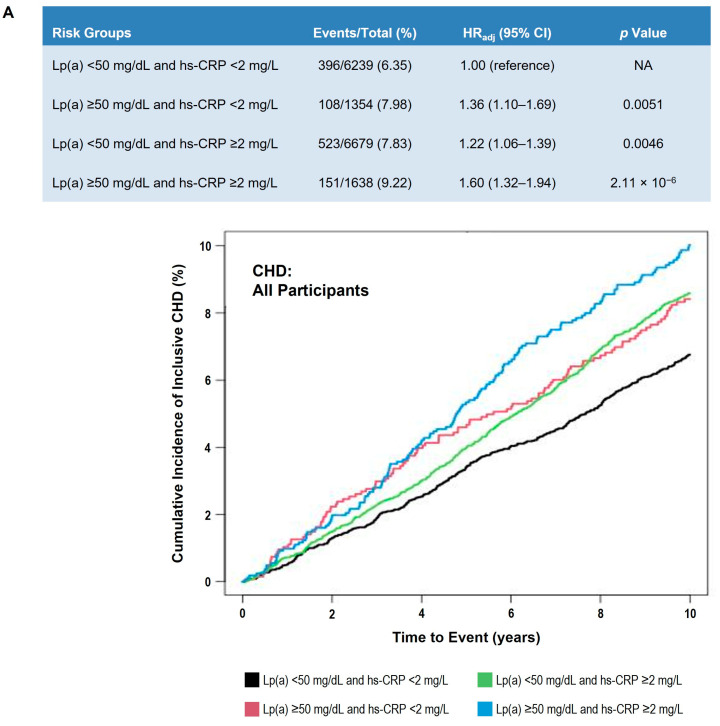

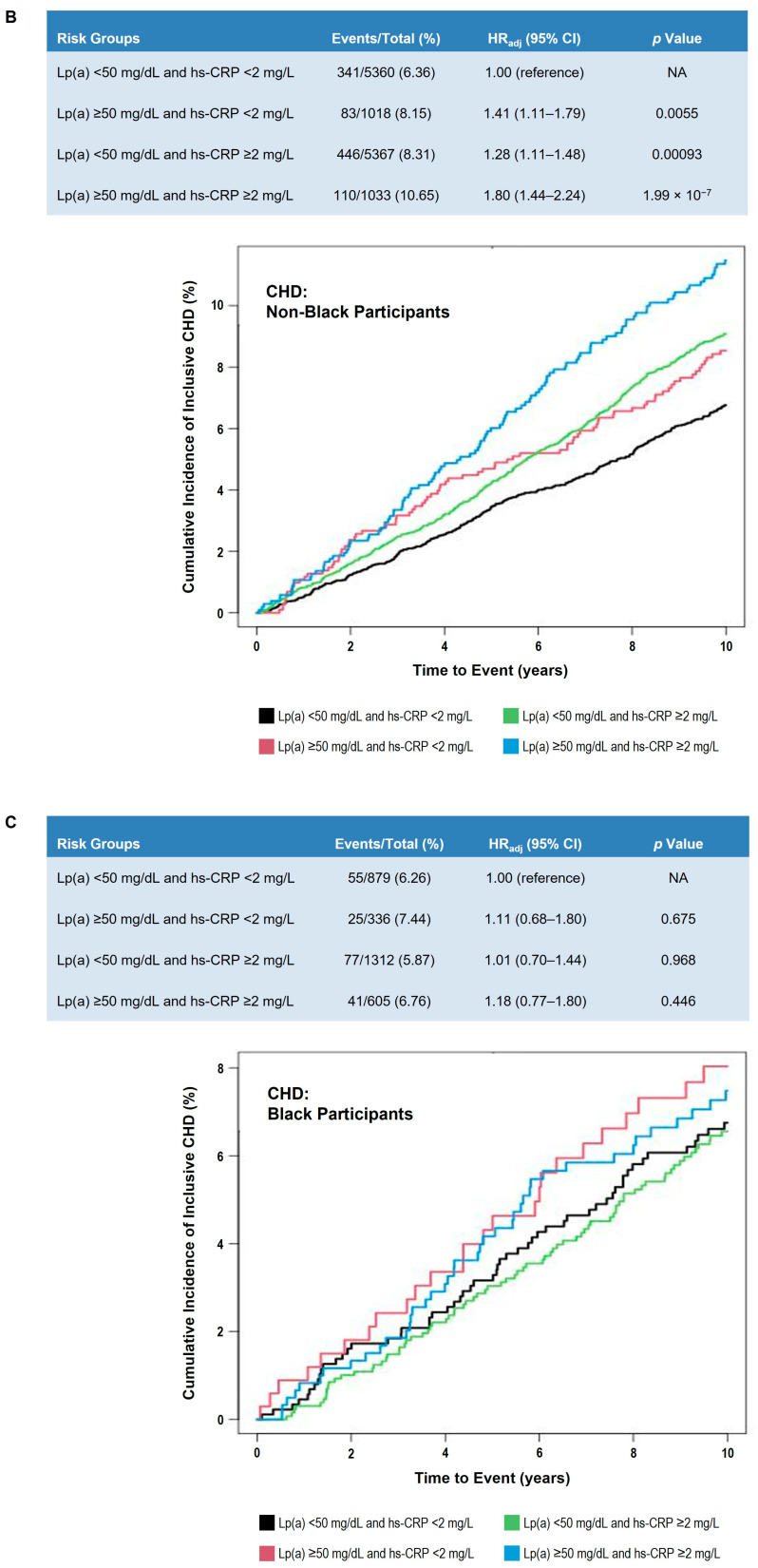
Adjusted 10-year CHD event risk in participants classified by Lp(a) and hs-CRP levels. (**A**) All participants. (**B**) Non-Black participants. (**C**) Black participants. Table shows number of events, HR_adj_ (95% CI), and *p* value by Lp(a) and hs-CRP clinical strata. Cox proportional hazards model was adjusted for age, sex, race (Panel **A** only), systolic blood pressure, hypertension treatment, smoking status, diabetes status, total cholesterol, high-density lipoprotein cholesterol, and cholesterol-lowering medication use. Lp(a) < 50 mg/dL and hs-CRP < 2 mg/L was used as the reference group. Graph presents Kaplan–Meier curves of cumulative incidence of events by prespecified Lp(a) and hs-CRP clinical strata. Black line indicates Lp(a) < 50 mg/dL and hs-CRP < 2 mg/L; red line shows Lp(a) ≥ 50 mg/dL and hs-CRP < 2 mg/L; green line shows Lp(a) < 50 mg/dL and hs-CRP ≥ 2 mg/L; blue line shows ≥50 mg/dL and hs-CRP ≥ 2 mg/dL. Abbreviations: CHD, coronary heart disease; CI, confidence interval; HR_adj_, adjusted hazard ratio; hs-CRP, high-sensitivity C-reactive protein; Lp(a), lipoprotein(a).

We also found similar results when we applied these stratified analyses to investigate the impact of hs-CRP on Lp(a)-associated CHD risk (Figure 5).

## 4. Discussion

In this combined analysis of the ARIC, FOS, and MESA cohorts, we found that elevated Lp(a) levels (≥50 mg/dL) were significantly associated with increased risk of incident ASCVD events and CHD events. The positive association of Lp(a) levels with ASCVD was mostly driven by CHD events rather than ischemic stroke events, was stronger in men and non-Black people, and was independent of diabetes status. Lp(a) levels ≥ 50 mg/dL were associated with increased 10-year ASCVD risk for individuals at intermediate risk (≥7.5% 10-year ASCVD risk). Although Lp(a) levels ≥ 50 mg/dL were associated with increased 10-year ASCVD risk in individuals at borderline risk (<7.5% 10-year ASCVD risk), the association was not statistically significant. Furthermore, we found a significant interaction between Lp(a) and hs-CRP, and individuals with concomitant elevated levels of Lp(a) and hs-CRP were at highest ASCVD risk.

Our main study finding showing that elevated Lp(a) levels predicted future ASCVD risk is consistent with data from previous population-based studies [2,16,17]. We found it somewhat surprising that we did not find a significant association of increased Lp(a) levels with risk of incident ischemic stroke in our study. However, prior data reported by large population-based studies on the relationship of Lp(a) levels and risk for stroke have been inconsistent, which may be partly attributable to differences in incident stroke subtypes and cohort composition, such as age, race, and time of follow-up [18,19,20,21]. Our finding that elevated Lp(a) levels were associated with increased ASCVD risk independent of diabetes status is consistent with previous reports [16,17].

In our study, elevated Lp(a) levels (≥50 mg/dL vs. <10 mg/dL) were associated with an even higher risk of incident ASCVD in individuals with diabetes compared with those without diabetes. Given the increased cardiovascular risk in diabetic individuals, measurement of Lp(a) levels may be particularly important for ASCVD risk stratification in individuals with prediabetes or diabetes [22].

The American Heart Association/American College of Cardiology guidelines on the primary prevention of ASCVD recommend the use of Lp(a) ≥ 50 mg/dL as a risk-enhancing factor to refine risk assessment in individuals aged 40–75 years who are at borderline or intermediate risk of ASCVD according to pooled cohort equations [9]. Our results show that Lp(a) levels ≥ 50 mg/dL were significantly associated with 10-year ASCVD risk in individuals at “intermediate ASCVD risk” (≥7.5% 10-year ASCVD risk). Furthermore, Lp(a) levels ≥ 30 mg/dL were also significantly associated with increased ASCVD risk in individuals at “intermediate ASCVD risk”. The utility of a single Lp(a) cut-off point rather than race-specific cut-off points for ASCVD risk prediction, as well as what specific Lp(a) cut-off point (e.g., 30 mg/dL, 50 mg/dL or 90th percentile of population) to use, are currently contested; no clear consensus has been reached on these issues.

As an inflammatory biomarker, hs-CRP is the most validated ASCVD risk predictor, and the 2019 American Heart Association/American College of Cardiology guidelines on the primary prevention of ASCVD recommend the use of hs-CRP as a risk-enhancing factor [9]. MESA investigators found a significant interaction between hs-CRP and Lp(a)-associated ASCVD risk, in which elevated Lp(a) levels were associated with primary ASCVD risk only in individuals with concomitant elevated hs-CRP levels (>2 mg/L) [12]. In our study, we also found a significant interaction between Lp(a) and hs-CRP, and individuals with concomitant elevated levels of Lp(a) and hs-CRP were at the highest risk of ASCVD. However, in contrast to the findings from MESA, we found that elevated Lp(a) levels were also associated with increased ASCVD risk in individuals with normal hs-CRP levels (<2 mg/L). The MESA population had fewer ASCVD events than our study of pooled cohorts and may have been underpowered for these stratified analyses, which may partly explain this apparent inconsistency between the two studies.

Our findings suggest that the interaction between hs-CRP and Lp(a)-associated ASCVD risk is quantitative (rather than qualitative) and that Lp(a)-associated ASCVD risk is exacerbated in a proinflammatory milieu. Indeed, it has been shown that the promotor region of the *LPA* gene contains five interleukin-6 response elements [23]. Furthermore, interleukin-6 blockade by the monoclonal antibodies tocilizumab inhibited apo(a) expression and Lp(a) synthesis in humans [24]. Whether interleukin-6 blockade could be a potential therapeutic option to treat elevated Lp(a) levels, particularly in individuals with chronic inflammation, needs further investigation.

It is known that there are interrelationships between the metabolism of apo(a) and apoB-100 within Lp(a) and VLDL apoB-100 metabolism, as well between VLDL-apoB-100 and hs-CRP metabolism [25,26]. While statins lower hs-CRP, they do not lower Lp(a), in contrast to proprotein convertase subtilisin kexin 9 inhibitors [27,28,29]. Apo(a) antisense therapy has been shown to be very effective in lowering Lp(a) levels [30,31], and this therapy is currently being investigated in clinical trials to determine its efficacy in lowering ASCVD risk in individuals with elevated serum Lp(a) levels. The data presented here indicate that such patients are at highest risk if they also have elevated hs-CRP levels.

Our study has several limitations, including the reliance on a single measurement of Lp(a) and hs-CRP, which can vary within individuals over time. We did not collect specific information on certain inflammatory diseases, which could result in residual confounding. As this is an observational study, the possibility of residual confounding cannot be eliminated and associations cannot be interpreted as causal. Our study also has a number of strengths. We leveraged the extensive data on cardiovascular risk factors, adjudicated ASCVD events, and biomarkers available from the combined cohorts of ARIC, FOS, and MESA with 10 years of follow-up time to investigate the efficacy of Lp(a) as an ASCVD-risk enhancer in a large multi-ethnic population of US adults. Furthermore, Lp(a) measurements were performed with the same Lp(a) assay, which has been shown to be less-sensitive to apo(a) isoform size, in the combined cohorts of ARIC, FOS, and MESA.

## 5. Conclusions

In the setting of primary prevention, elevated Lp(a) levels were associated with increased ASCVD risk, particularly in individuals with concomitant elevated hs-CRP levels and those at intermediate 10-year ASCVD risk. Clinical trials using apo(a) antisense and interleukin-6 blockade therapies are needed to show their potential efficacy in lowering Lp(a) levels and reducing ASCVD risk in individuals with elevated Lp(a) and chronic inflammation.

## Figures and Tables

**Table 1:** Baseline characteristics of study participants stratified by Lp(a) quintiles.

Characteristics	Quintile 1 2.5 [1.5–3.5]	Quintile 2 7.4 [6.0–8.7]	Quintile 3 14.7 [12.3–17.5]	Quintile 4 31.5 [25.4–39.0]	Quintile 5 70.4 [57.6–90.5]	*p*-Value
Age (years)	61 (11)	61 (11)	62 (11)	62 (12)	61 (11)	0.385
Males	1623 (47.3)	1591 (46.4)	1548 (44.9)	1472 (42.9)	1287 (37.3)	<0.001
Females	1811 (52.7)	1840 (53.6)	1902 (55.1)	1957 (57.1)	2160 (62.7)	<0.001
Non-Blacks	3283 (95.6)	3166 (92.3)	2847 (82.5)	2126 (62.0)	2336 (67.8)	<0.001
Blacks	151 (4.4)	265 (7.7)	603 (17.5)	1303 (38.0)	1111 (32.2)	<0.001
Systolic BP (mm Hg)	124 (24)	123 (24)	124 (26)	126 (26)	125 (27)	<0.001
Diastolic BP (mm Hg)	71 (14)	70 (13)	72 (14)	72 (14)	72 (14)	<0.001
Antihypertensive use	1199 (34.9)	1121 (32.7)	1147 (33.2)	1366 (39.8)	1326 (38.5)	<0.001
Diabetes	452 (13.2)	373 (10.9)	333 (9.7)	438 (12.8)	432 (12.5)	0.711
Diabetes medication use	200 (5.8)	194 (5.7)	183 (5.3)	260 (7.6)	267 (7.7)	<0.001
Body weight (lbs)	173 (51)	173 (52)	172 (51)	174 (51)	172 (51)	0.002
Waist circumference (cm)	100 (18)	99 (18)	98 (18)	99 (19)	98 (19)	<0.001
Smoking	457 (13.3)	509 (14.8)	517 (15.0)	501 (14.6)	480 (13.9)	0.595
Total cholesterol (mg/dL)	194 (45)	195 (45)	197 (46)	199 (46)	208 (46)	<0.001
HDL-cholesterol (mg/dL)	46 (22)	47 (20)	48 (19)	49 (20)	50 (20)	<0.001
Triglycerides (mg/dL)	130 [92–190]	123 [87–174]	114 [83–162]	109 [79–154]	110 [80–153]	<0.001
LDL-cholesterol (mg/dL)	115 (41)	118 (41)	121 (41)	123 (44)	130 (41)	<0.001
Statin use	259 (7.5)	216 (6.3)	258 (7.5)	255 (7.4)	381 (11.1)	<0.001
hs-CRP (mg/L)	2.1 [1.0–4.8]	2.0 [0.9–4.6]	2.1 [1.0–4.9]	2.4 [1.0–5.1]	2.4 [1.1–5.4]	<0.001

Values presented are number (proportion) for categorical variables, mean (SD) for variables normally distributed, median [25th–75th percentile] for variables not normally distributed. Abbreviations: BP, blood pressure; HDL, high-density lipoprotein; hs-CRP, high sensitivity C-reactive protein; LDL, low-density lipoprotein; Lp(a), lipoprotein(a)

**Table 2:** Characteristics of subjects at baseline by event outcome.

	No Event (*n* = 14,385)	ASCVD (*n* = 1548)			CHD (*n* = 1168)		
	Value	Value	HR (95% CI)	*p* Value	Value	HR (95% CI)	*p* Value
DEMOGRAPHICS							
Females, *n* (%)	8535 (58.5)	572 (36.8)	0.41 (0.37–0.45)	1.59 × 10^−64^	382 (32.8)	0.34 (0.30–0.39)	1.01 × 10^−66^
Age, year	61 (56–67)	65 (59–69)	1.83 (1.70–1.98)	1.59 × 10^−57^	64 (59–69)	1.74 (1.56–1.89)	6.01 × 10^−37^
Black, *n* (%)	2889 (19.9)	305 (19.6)	1.01 (0.89–1.14)	0.888	196 (16.7)	0.83 (0.72–0.97)	0.020
Non-Black, *n* (%)	11,623 (80.1)	1248 (80.4)	0.99 (0.87–1.12)	0.888	976 (83.3)	1.20 (1.03–1.40)	0.020
CLINICAL/TREATMENT							
Systolic BP, mmHg	123 (112–137)	130 (118–145)	1.53 (1.44–1.62)	3.21 × 10^−47^	129 (117–143)	1.42 (1.33–1.52)	2.93 × 10^−24^
BP Rx, *n* (%)	4912 (33.8)	790 (48.9)	1.90 (1.72–2.10)	7.67 × 10^−37^	565 (48.2)	1.87 (1.66–2.09)	1.45 × 10^−26^
Diabetes, *n* (%)	1540 (10.6)	308 (19.8)	2.19 (1.94–2.49)	5.82 × 10^−35^	233 (19.9)	2.22 (1.92–2.56)	1.79 × 10^−27^
Diabetes Rx, *n* (%)	825 (5.7)	182 (11.7)	2.42 (2.07–2.82)	6.82 × 10^−29^	138 (11.8)	2.44 (2.04–2.92)	9.04 × 10^−23^
Cholesterol-lowering Rx, *n* (%)	1066 (7.3)	188 (12.1)	1.70 (1.46–1.98)	9.99 × 10^−12^	152 (13.0)	1.84 (1.55–2.18)	2.11 × 10^−12^
Smoking, *n* (%)	2030 (14.0)	291 (18.7)	1.48 (1.30–1.68)	1.50 × 10^−9^	215 (18.3)	1.42 (0.25–1.68)	7.71 × 10^−7^
LIPIDS							
Total cholesterol, mg/dL	198 (177–223)	201 (178–225)	1.07 (1.10–1.14)	0.046	202 (178–227)	1.11 (1.03–1.19)	0.0065
Triglycerides, mg/dL	115 (82–164)	133 (94–186)	1.13 (1.10–1.16)	1.31 × 10^−16^	136 (95–188)	1.14 (1.10–1.17)	1.09 × 10^−15^
LDL-C, calculated, mg/dL *	121 (101–142)	125 (105–148)	1.19 (1.12–1.27)	2.08 × 10^−8^	127 (108–151)	1.27 (1.19–1.36)	1.84 × 10^−11^
HDL-C, mg/dL	49 (40–61)	42 (36–53)	0.59 (0.55–0.64)	7.62 × 10^−44^	42 (35–52)	0.52 (0.47–0.56)	6.00 × 10^−48^
Non-HDL-C, mg/dL ^†^	147 (124–171)	155 (132–180)	1.28 (1.20–1.35)	9.35 × 10^−16^	157 (133–182)	1.36 (1.27–1.45)	1.03 × 10^−19^
Lp(a), mg/dL continuous	14.5 (6.0–38.3)	15.6 (6.3–44.0)	1.08 (1.03–1.14)	0.0018	16.4 (6.4–45.0)	1.11 (1.05–1.17)	0.00031
Lp(a) ≥ 30 mg/dL, *n* (%)	4420 (30.5)	527 (33.9)	1.16 (1.05–1.29)	0.0051	414 (35.3)	1.24 (1.0–1.39)	0.00052
Lp(a) ≥ 50 mg/dL, *n* (%)	2684 (18.5)	328 (21.1)	1.17 (1.04–1.32)	0.012	256 (21.8)	1.22 (1.06–1.40)	0.0045
Lp(a) ≥ 90th percentile, *n* (%)	1412 (9.7)	176 (11.3)	1.18 (1.01–1.38)	0.037	134 (11.4)	1.20 (1.00–1.43)	0.049
INFLAMMATION							
hs-CRP, mg/L	2.10 (0.95–4.84)	2.60 (1.17–5.72)	1.05 (1.03–1.07)	2.55 × 10^−6^	2.50 (1.13–5.38)	1.04 (1.01–1.07)	0.020

Values are median (25th–75th percentile) for continuous variables or number (%) for categorical variables. Hazard ratios for continuous variables represent comparison across interquartile range, the 75th percentile vs. the 25th percentile, with no event as reference. Variables not normally distributed were log-transformed prior to regression analysis. * Value is calculated using the Friedewald equation: (total cholesterol − HDL-C − TG/5). ^†^ Value is calculated using the following equation: total cholesterol − HDL-C. Abbreviations: ASCVD, atherosclerotic cardiovascular disease; BP, blood pressure; CI, confidence interval; HDL-C, high-density lipoprotein cholesterol; HR, hazard ratio; hs-CRP, high sensitivity C reactive protein; LDL-C, low-density lipoprotein cholesterol; Lp(a), lipoprotein(a); no event, subjects who had no event or procedure prior to baseline and no event or procedure during 10-year follow-up; Rx, treatment.

## Data Availability

The dataset presented in this study may be available on request from the corresponding author. Limitations may apply due to legal reasons. This is a pooling data analysis from three studies; investigators wishing to receive and analyze data from any of these studies should contact the research committee of each study for approval.

## Supplementary Materials

The following supporting information can be downloaded at: https://www.mdpi.com/article/10.3390/nu17081324/s1, Supplemental Table S1. Sex and ethnic demographics of subjects by event outcome.

## Abbreviations

Apo	apolipoprotein
ARIC	Atherosclerosis Risk in Communities Study
ASCVD	atherosclerotic cardiovascular disease
CHD	coronary heart disease
CI	confidence interval
FOS	Framingham Offspring Study
HDL-C	high-density lipoprotein cholesterol
HR	hazard ratio
hs-CRP	high sensitivity C-reactive protein
LDL-C	low-density lipoprotein cholesterol
Lp(a)	lipoprotein(a)
MESA	Multi-Ethnic Study of Atherosclerosis
TG	triglycerides
