# Supplementary material for: Lipoprotein(a) and Risk of Incident Atherosclerotic Cardiovascular Disease: Impact of High-Sensitivity C-Reactive Protein and Risk Variability Among Human Clinical Subgroups
Source: Nutrients. 2025 Apr 11;17(8):1324. doi: 10.3390/nu17081324 (PMC12030245; doi:10.3390/nu17081324)
Supplement: Supplementary file 1 [file nutrients-17-01324-s001.zip › nutrients-3478688-supplementary.pdf]

SUPPLEMENTAL Table S1. Sex and ethnicity demographics of participants by event outcome

	No Event (n = 14,385)	ASCVD (n = 1,548)	CHD (n = 1,168)	Ischemic Stroke (n = 335)
Males*	5,915 (41.1)	980 (63.3)	788 (67.5)	181 (54.0)
Females*	8,470 (58.9)	568 (36.7)	380 (32.5)	154 (46.0)
Black*	2,839 (19.7)	302 (19.5)	193 (16.5)	100 (29.9)
Non-Black*	11,546 (80.3)	1,246 (80.5)	975 (83.5)	235 (70.1)
European ancestry†	10,160 (88.0)	1,146 (92.0)	910 (93.3)	209 (88.9)
Hispanic/Latino ancestry†	876 (7.6)	75 (6.0)	47 (4.8)	23 (9.8)
Asian or other ancestry†	510 (4.4)	25 (2.0)	18 (1.8)	3 (1.3)

*Data presented as n (%) of cohort group.

†Data presented as n (%) of total number of non-Blacks.

ASCVD, atherosclerotic cardiovascular disease; CHD, coronary heart disease.
